# Digital Workflow for Edentulous Patients with Implant-Supported Fixed Prostheses: A Fully Digital Technique

**DOI:** 10.3390/dj10090174

**Published:** 2022-09-15

**Authors:** Seung Wook Jung, Yi-Qin Fan, Chunui Lee

**Affiliations:** Department of Oral and Maxillofacial Surgery, Wonju College of Medicine, Yonsei University, Wonju 26426, Korea

**Keywords:** digital workflow, CAD/CAM, digital implant prosthetic, edentulous

## Abstract

Dentists have made prostheses using traditional methods, which are inconvenient and time-consuming. It includes functional impression taking, plaster model production, wax rim production, intermaxillary relationship and occlusal plane setting, artificial tooth arrangement, denture polymerization, polishing, etc. To make prostheses in this way, the patient has to visit the dentist several times, and it takes a long time for them to receive treatment. In addition, the potential for errors associated with the denture-manufacturing process and the use of denture materials has always existed. However, the recent use of digital technology in dentistry has made it possible to create digital prostheses. Several techniques for the immediate loading of implants with a fixed prostheses in edentulous patients have been developed. However, these techniques are partially digital techniques that include laboratory work for prosthesis fabrication. This article aimed to describe a fully digital technique for implant-supported fixed prostheses. It includes intra-oral scanning of edentulous patients, implant placement planning, and final prosthesis fabrication. This technique facilitates a simple and more efficient immediate restoration after implant placement without using stone casts.

## 1. Introduction

The analog system for making prostheses using implants for edentulous patients involves taking impressions, making plaster models, and manual fabrication. In contrast, digital systems consist of scanned images and computer-aided design and computer-aided manufacture (CAD/CAM). By using a digital system in the treatment of edentulous patients, the first step is to take a digital impression, and the second step is to assess the intermaxillary relationship. When the digital impression and intermaxillary relationship are acquired, an edentulous image of a three-dimensional image with an intermaxillary relationship is obtained, thus making digital prostheses possible. With the development of digital technology, researchers have tried various methods for manufacturing implants and dentures using these digital technologies [1,2,3]. Digital technology is currently used to solve problems and inconveniences experienced with conventional methods during the diagnostic work process and the long treatment time. Immediate loading of implants with a fixed prosthesis in edentulous patients has now become an established and viable treatment concept [1,3].

The decision regarding the placement of an immediate final prosthesis or an immediate provisional prosthesis is determined by factors such as cost, preoperative time commitments, postoperative commitments, laboratory support, and patient considerations [1].

Provisional prostheses made of resin or metal-reinforced resin have been fabricated previously [2,4,5,6]. Alternatively, the existing denture has been transformed into a provisional prosthesis by connecting temporary cylinders on the implants and holes in the denture by luting with acrylic resin [7,8]. The immediate insertion of the definitive fixed prosthesis after the placement of implants has also been reported [9,10,11,12,13,14]. However, these methods are partially digital techniques that include laboratory work for prosthesis fabrication. Thus, this study presents a fully digital technique for implant-prosthetic rehabilitation starting from acquiring the occlusal vertical dimension (OVD) and planning implant placement up to the immediate insertion of the definitive prosthesis. Previous methods of obtaining OVD involve a cumbersome process of fabricating, recording bases, and wax rims. However, scanning immediate dentures or used dentures to obtain the patient’s OVD is convenient for the patient, saves time, and solves problems without laboratory work. In addition, it is easy and convenient to obtain an image of the edentulous gingiva and intraoral information because the denture can be scanned outside the oral cavity.

## 2. Materials and Methods

Among the edentulous patients who came to our hospital, Wonju Severance Christian Hospital, patients who visited for the fabrication of an implant-supported fixed prosthesis were treated using digital technology to install temporary prostheses and even final prostheses.

The study protocol was reviewed and approved by the Institutional Review Board of Yonsei University Wonju Severance Christian Hospital, Wonju, Korea (CR 316127). The case described herein is of a 76-year-old male patient with a completely edentulous maxilla. He visited our hospital for implant-supported fixed prosthetic treatment because of the discomfort of the existing dentures. He agreed to present his case in this paper and signed the consent form. His medical history was hypertension, and he was taking antihypertensive drugs.

For imaging, cone-beam computed tomography (CBCT) Point 3D Combi 500C (PointNix, Seoul, South Korea) was used. An intraoral scanner (TRIOS 3; 3Shape A/S, Copenhagen, Denmark), implant planning software (Implant Studio; 3Shape A/S, 2015, Copenhagen, Denmark), 3D printer for surgical guide (Probe; DIO Inc., Pusan, South Korea), and CAD/CAM milling process machine (ARUM 5X-450, Doowon ID, Daejoen, South Korea) were used. Treatment was performed by the digital flow method, as described below:

### Technical Description

**Figure 1:**
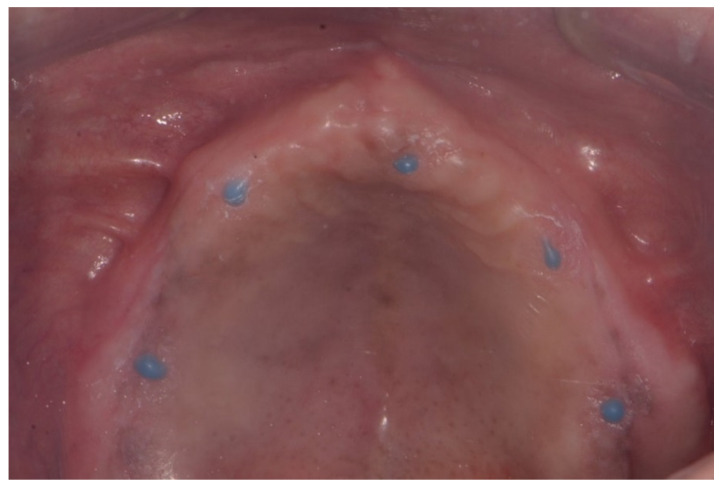
Radiopaque and flowable composite resin on the maxillary mucosa.

**Figure 2:**
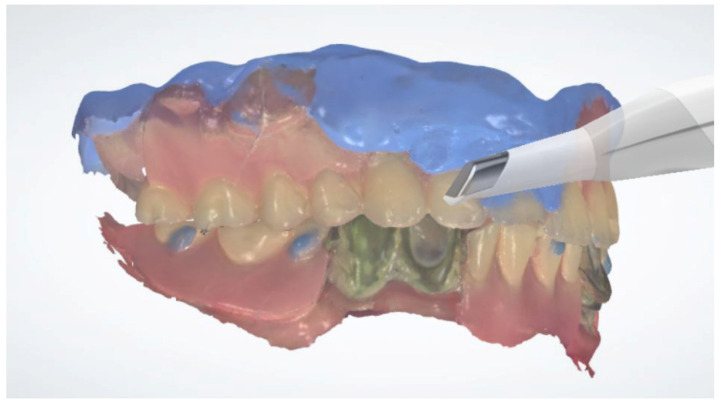
Scan the impression surface of the patient’s prostheses. The upper and lower occlusal relationships were scanned.

**Figure 3:**
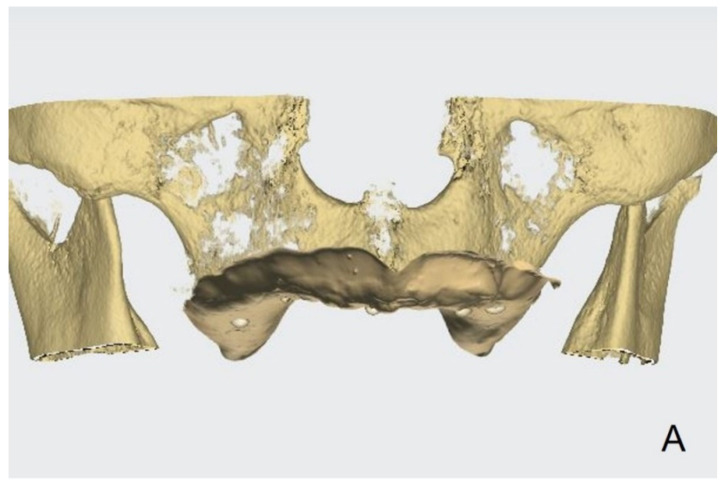

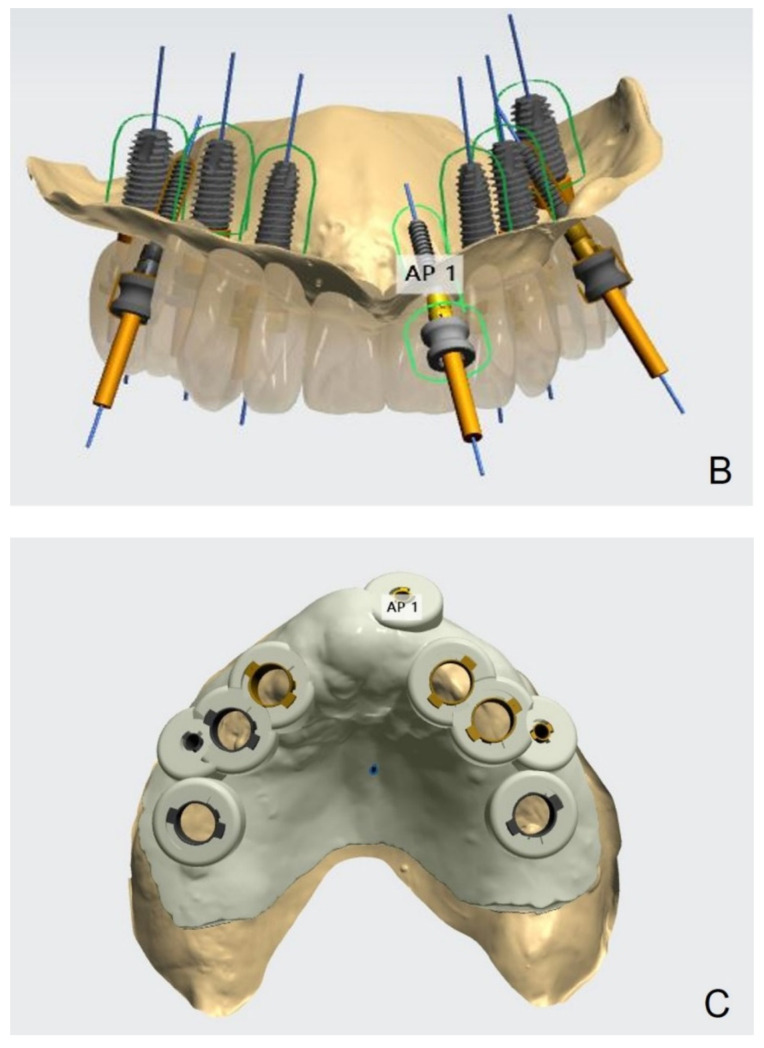
(**A**) Image fusion by matching resin markers present on the intraoral scan and CBCT. (**B**) Virtual implant planning. (**C**) Designed guide for the placement of six dental implants.

**Figure 4:**
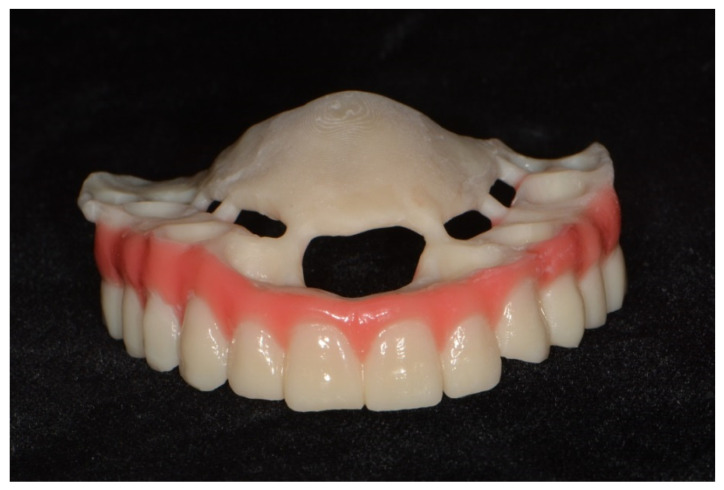
Interim restoration with tissue-colored composite resin on the gingival area.

**Figure 5:**
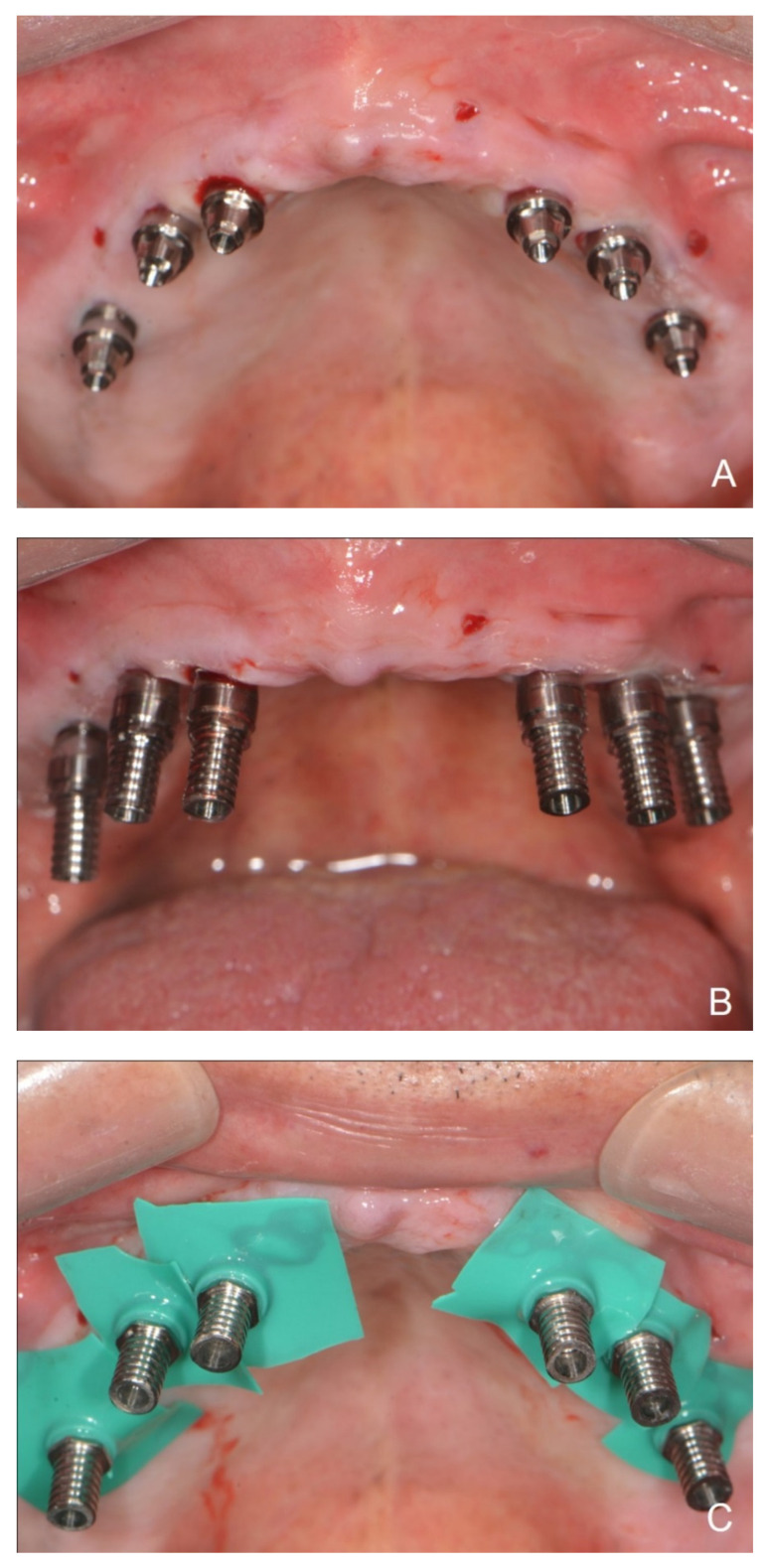

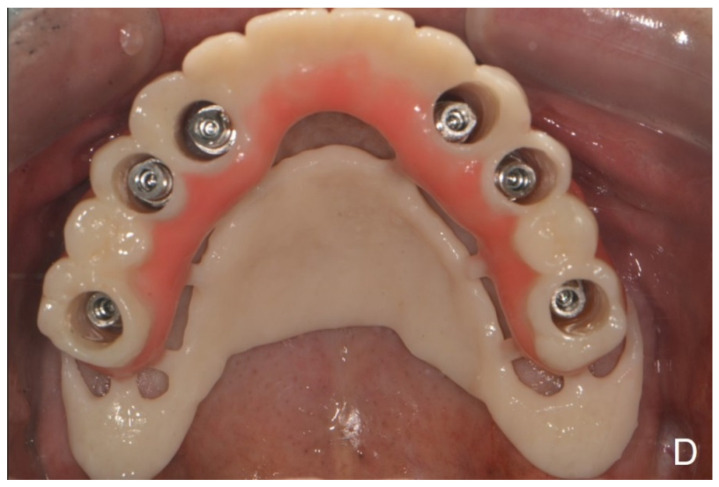
(**A**) Abutments in place. (**B**) Connection of the interim cylinders to the abutments. (**C**) Rubber dam placed beneath the interim cylinders to cover and protect the underlying soft tissue. (**D**) Evaluation of the interim restoration over interim cylinders.

**Figure 6:**
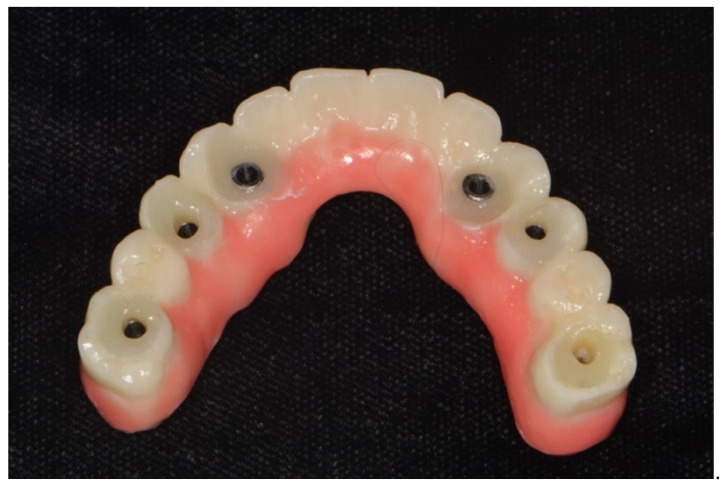
Interim prosthesis after sectioning the denture flange part.

**Figure 7:**
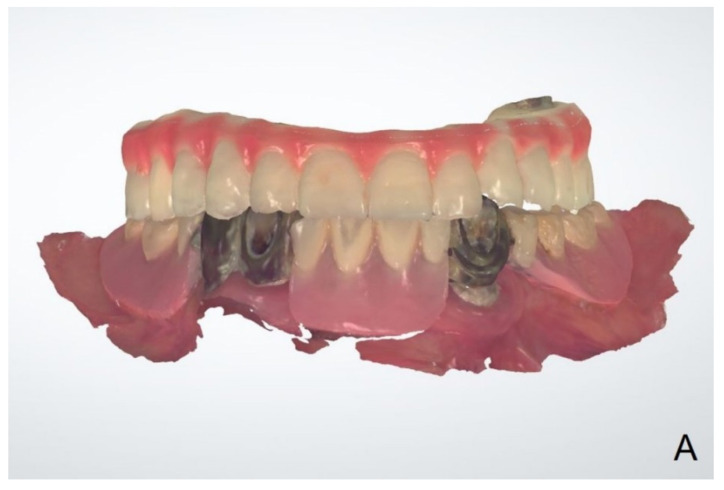

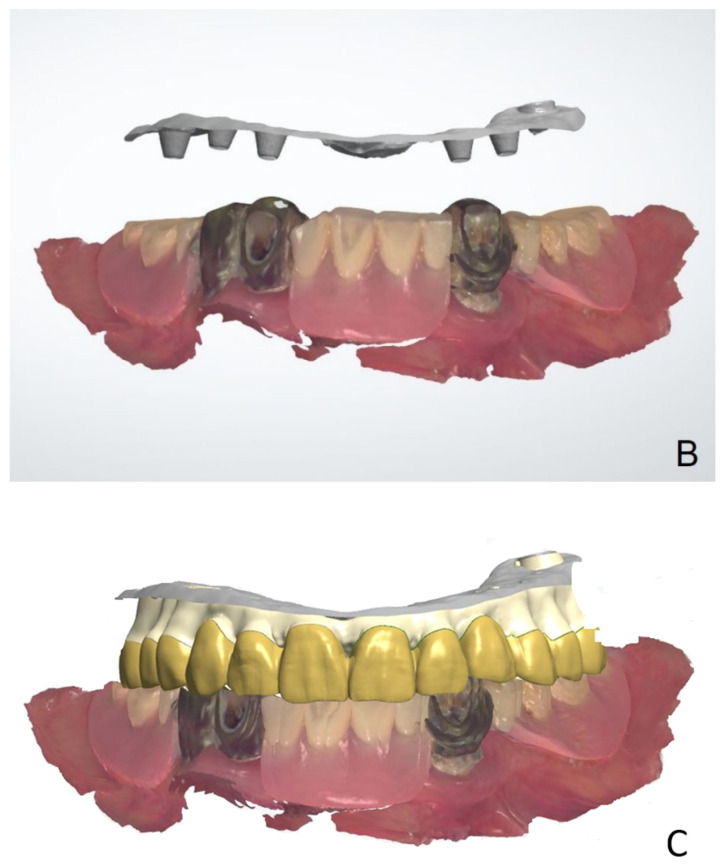
(**A**) Scan of the interim prosthesis. (**B**) Digital model of the cylinder cap and interim prosthesis base with the opposing teeth mounted at the occlusal vertical dimension. (**C**) Virtual metal framework and crown designed by using the virtual design software.

**Figure 8:**
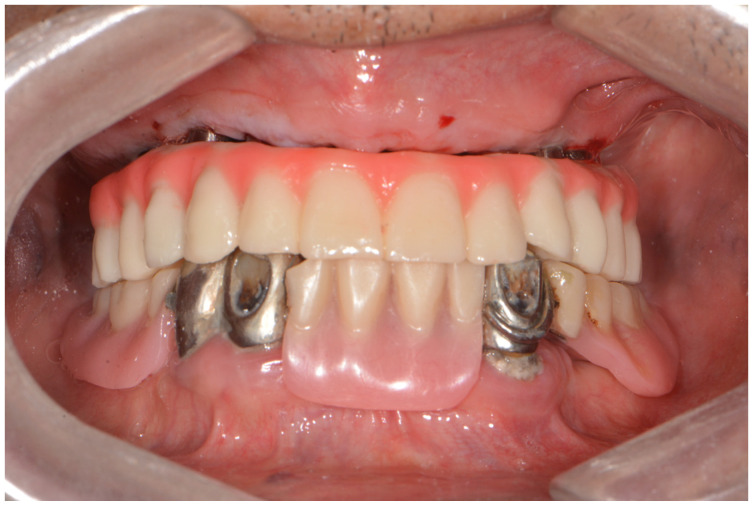
Definitive restoration fabricated by CAD/CAM based on direct digital scans of the interim restorations made with intraoral scanners.

1. Inject radiopaque flowable composite resins (Charmfil Flow; Dentkist Inc., Seoul, Korea) in hemispheres of 1–2 mm diameter in ≥3 sites of the attached gingiva (Figure 1). These pose as markers for superimposing intraoral scans and CBCT data. After photopolymerization of the injected resin, apply tissue adhesive (Histoacryl; B. Braun, Aesculap AG, Tuttlingen, Germany) around the resin marker to prevent the resin from falling off.
2. Place vinyl polysiloxane with the light viscosity consistency of the material inside the patient’s existing denture and obtain an impression in occlusion similar to that using a custom tray. If the resin marker is attached intraorally, an impression of the resin marker is also obtained.
3. Obtain CBCT images of the maxilla and mandible with the resin markers using a Point 3D Combi 500C (PointNix).
4. Scan the impression surface of the denture, opposing teeth, and bite, registering the relationship of the jaw with the denture using an intraoral scanner (TRIOS 3; 3Shape A/S) (Figure 2).
5. Obtain the tissue surface details by scanning the impression surface of the denture with the intraoral scanner. Import the DICOM data of the inverted scan images and CBCT images into an implant-planning software (Implant Studio; 3Shape A/S, 2015). Virtually merge the inverted scan images and CBCT images by matching the hemisphere resin markers present in both (Figure 3A).
6. Following image superimposition, plan the positions for the six implants based on the virtual tooth arrangement (Figure 3B). Subsequently, print the surgical guide for implant placement using a 3D printer (Probe; DIO Inc., Pusan, South Korea) (Figure 3C). Design an interim prosthesis for immediate loading after implant surgery using a virtual design program (Dental Designer; 3Shape A/S). The interim prosthesis consists of two parts—a fixed part similar to the definitive restoration and a denture flange part for repositioning the fixed part in the oral cavity (Figure 4). The flange part should be easily separable from the fixed part. After the design is complete, 3D-print the interim prosthesis using a commercial printable resin (DIOnavi Crown&Bridge; DIO Inc.).
7. After implant placement, remove the surgical guide and connect the abutment to the implant (Figure 5A). Then, connect the interim cylinder to the abutment (Figure 5B). Connect the interim prosthesis to the interim cylinder by adding acrylic resin (Duo-Link; Bisco Dental, Schaumburg, IL, USA) to the space between them using a syringe (Figure 5C,D). After allowing the resin to polymerize, remove the flange part of the interim prosthesis (Figure 6). Screw the attached part of the prosthesis to the abutment. Check for occlusal interferences and make occlusal adjustments if necessary.
8. Scan all sides of the interim prosthesis extraorally using the intraoral scanner to obtain scan data. With the interim prosthesis in the mouth, scan its labial surface, opposing teeth, and occlusal relationship using the intraoral scanner (Figure 7A).
9. Reverse the interim prosthesis image using a CAD software (Shape Designer; 3Shape A/S) to obtain a positive image from the negatively recorded inside image of the cylinder (Figure 7B). Superimpose the markers on the cylinder cap and reversed cylinder images. Based on the previously registered occlusion, obtain a digital model of the cylinder cap and the interim prosthesis base associated with the opposing teeth.
10. Design a virtual metal framework and crowns using the virtual design software (Dental Designer; 3Shape A/S) (Figure 7C). Subsequently, fabricate the definitive restoration by using the CAD/CAM milling process (ARUM 5X-450; Doowon ID) (Figure 8).

In this case, six implants were placed in positions #13, 14, 16, 23, 24, and 26 to fabricate a fixed prosthesis with an all-on-6 screw-retained implant in the maxilla. The sizes of each implant were as follows: #13i, 23i: 3.8 mm × 10 mm; #14i, 24i: 4.5 mm × 10 mm; and #16i, 26i: 5.0 mm × 10 mm.

For patients who need a tooth extraction, the OVD is collected and stored using an oral scanner before tooth extraction. When the OVD is lowered because of tooth loss, residual tooth mobility, or tooth abrasion, dentures can be manufactured by acquiring proper OVD by simulating the mandibular movement using the virtual articulator on CAD software.

## 3. Results

**Figure 9:**
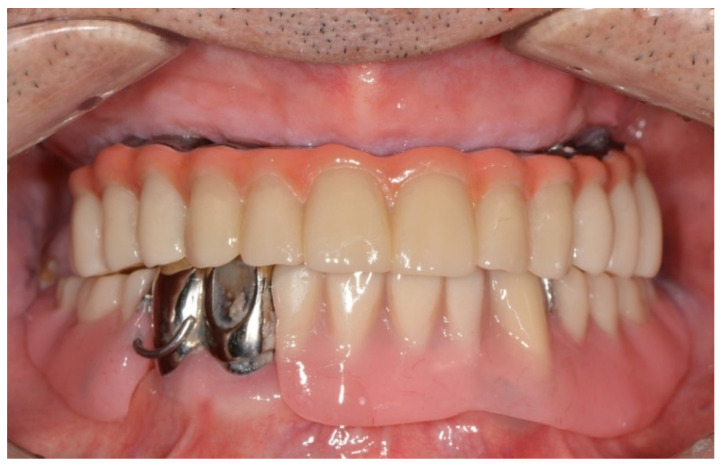
After extraction of tooth #33, the mandibular prosthesis immediately completed by CAD/CAM using the patient’s existing 3D data.

The patient showed great satisfaction with the treatment made by placing the implant and making a temporary prosthesis immediately on the day of the visit. When implant placement and denture installation were performed separately, the patient could hardly masticate, and there may have been significant esthetic deficits. However, by using digital flow, it was possible to increase patient satisfaction and shorten the treatment time. The patient came to the hospital 1 week after treatment, and the occlusal status was confirmed. The occlusion state was good, and no special problems in mastication function occurred. The patient was also satisfied with the esthetic aspect. After 2 weeks, he visited the hospital again, and through an extraoral scan of the temporary prosthesis, the final prosthesis was fabricated with a titanium frame and inserted into the patient’s mouth. At his visit 6 months later, the mobility of tooth #33 was severe, and it was extracted. Using the patient’s existing 3D data, the mandibular prosthesis was also immediately completed (Figure 9).

## 4. Discussion

This report aimed to describe a fully digital technique for implant-supported prostheses in edentulous patients. Patients who are using temporary dentures or prostheses at the initial visit can use and store the information obtained through the oral scanner. Moreover, for patients who need a tooth extraction, OVD can be obtained using the oral scanner before a tooth extraction, and patient’s data can be stored. As in the presented case, using the patient’s data, it is possible to easily manufacture prostheses by maintaining the OVD even during mandibular treatment after maxilla treatment is finished. The interim prosthesis is digitally designed using CBCT scan data and a digital impression of the patient’s oral cavity obtained through an intraoral scanner and fabricated through 3D printing [15]. The interim prosthesis has a fixed component that has cylinder access holes and a denture flange part that is designed to be easily cut off.

The definitive prosthesis is fabricated by the CAD/CAM technique based on a direct digital impression of the interim prosthesis using an intraoral scanner. This technique allows clinicians to construct the prosthesis quickly and efficiently in 2 days following implant placement without using stone casts. An immediate definitive prosthesis decreases the number of visits and treatment duration [16,17]. In addition, the esthetics, occlusion, and vertical dimension can be scrutinized through the interim prosthesis; thus, modification is easy before the fabrication of the definitive prosthesis. Therefore, the precision of the interim prosthesis is a key factor in fabricating a successful, accurate definitive prosthesis [18]. A precise fit of the cylinder with the abutment without any gap between them is essential. In the absence of a perfect fit between both components, the cylinder must be reconnected.

Studies have shown that digital impressions are as accurate and clinically successful as conventional impressions [19,20,21]. Before the remaining teeth are extracted and the jaw becomes edentulous, many patients have severe tooth decay or advanced periodontitis, which results in severe tooth mobility. In this case, the traditional impression-taking method will be extracted together with the impression materials. This problem is not encountered in digital impression taking because digital images are obtained without taking impressions.

Conventionally, interim prostheses have been fabricated from laboratory-processed polymethyl methacrylate; however, there are reports of fractures in these prostheses [22,23,24,25,26]. Despite improvements in restorative materials and techniques, fractures of interim prostheses are a common complication, which may result in a loss of the implant because of harmful forces [27,28,29,30,31]. This problem can be avoided by the immediate insertion of a strong and stable definitive prosthesis after implant placement, which may aid in osseointegration of the implant.

The prosthesis used in this technique was a screw-retained type, which facilitated the removal and scanning of the interim prosthesis to fabricate the definitive prosthesis. A cement-retained prosthesis would not allow for the scanning of the interim prosthesis. Primary implant stability is considered a key requirement for immediate loading. Most studies have reported that a minimum implant insertion torque of 30–35 Ncm is required for immediate loading [16,23,26,32,33,34,35,36,37]. In this technique, the implant insertion torque to loosen the screw and detach the interim prosthesis was >35 Ncm. If the implant insertion torque is <35 Ncm, the current technique is not possible because the interim prosthesis cannot be removed. This is a constraint of this technique.

Another limitation of this technique is that errors can occur due to recording impressions from the patient’s existing dentures. Moreover, attaching resin markers in the oral cavity is difficult when there is little immobile mucosa, especially in patients with an atrophic mandible. In these cases, a resin marker may be affixed to the patient’s existing denture. However, dentures with metal frameworks generate artifacts during CBCT [38]. Additionally, implants may be positioned differently from planned, if the surgical guide is not properly positioned. Moreover, it may not be possible to position passively through the cylinder access holes of the interim prosthesis, which take longer to adjust [39].

In this technique, digital impression taking with an oral scanner is a basic and very important process. For accurate digital impression taking, the use of a retractor with a frame and handle made of aluminum wire when scanning is recommended. If the tissue in the oral cavity moves, the scanning is not performed accurately; thus, using a retractor can fix the moving tissue. When scanning the maxillary edentulous jaw, scan the alveolar ridge and the palate together in a “W” shape from left to right and scan the wide palate after attaching a marker to reduce errors. Mandibular edentulous jaw scan is performed similarly, but it should be controlled by retracting the buccal mucosa and tongue. If an edentulous patient does not have a denture, there is a limit to finding a centric position or acquiring an OVD using digital techniques alone. In this situation, clinicians should use wax rim dentures or a gothic arch tracer to obtain objective information about vertical height and centric position. If image information such as the patient’s smile line and lip support can be reflected and TMJ movement can be accurately reproduced on a computer using a digital sensor, digital prostheses can be produced even in patients with collapsed OVD.

## 5. Conclusions

This article presents a fully digital technique for implant-supported prosthesis rehabilitation, from acquiring OVD and planning implant placement to the immediate insertion of the definitive prosthesis in edentulous patients. This technique allows for a simple and more efficient immediate restoration after implant placement without using stone casts. It also decreases the number of visits and treatment duration and gives great satisfaction to the patient.

## Data Availability

Data are available upon reasonable request.
